# Early Intervention for Psychosis in emerging countries: findings from a first-episode psychosis programme in Ribeirão Preto, Brazil

**DOI:** 10.1192/j.eurpsy.2022.798

**Published:** 2022-09-01

**Authors:** G. Correa-Oliveira, L. Scarabelot, J. Morais Araujo, A. Boin, R. Mendes Paula Pessoa, L. Rodrigues Leal, C. Del-Ben

**Affiliations:** Ribeirão Preto Medical School, University of São Paulo, Neuroscience And Behaviour, Ribeirão Preto, Brazil

**Keywords:** early intervention, PSYCHOTIC DISORDERS, latin america, First-episode psychosis

## Abstract

**Introduction:**

People presenting first-episode psychosis (FEP) benefit from early intervention programmes, although they are scarce in low- and middle-income countries (LMICs). In Brazil, there are just a few of them unequally distributed across the country.

**Objectives:**

We aimed to describe the workings of the Ribeirão Preto Early Intervention for Psychosis Programme (Ribeirão Preto-EIP) – an outpatient service for first-episode psychosis patients residents in the Ribeirão Preto catchment area in Southeastern Brazil.

**Methods:**

A retrospective cohort of all patients attended throughout four years (2015-2018) was analysed. We excluded patients who attended only the first consultation and those with an initial diagnosis other than a psychotic disorder. Data was obtained through retrospective analysis of medical records.

**Results:**

Our service had 358 new referrals during the four-year period, and 237 patients were followed on average (median) by 14 months. Most of the patients were male (64.1%), single (84.8%), with a median age of 23.5 years (age ranged from 9 to 86 years). Schizophrenia was the main diagnosis (43.4%), followed by substance-induced (25.7%) and affective psychosis (18.6%). Taking follow-up diagnoses as gold-standard, initial diagnoses of bipolar disorder and schizophrenia spectrum disorders had the highest positive predictive values, 83% and 81% respectively. Most referrals to our programme were made by tertiary care (63.7%), followed by secondary (28.5%) and primary care (7.8%).

**Conclusions:**

Here we presented a large sample of FEP patients in a representation as trustworthy to the reality of our programme as possible. Our analysis suggest that Early Intervention Programmes can be successfully implemented in LMICs.

**Disclosure:**

No significant relationships.

